# Generative Adversarial Network Combined with SE-ResNet and Dilated Inception Block for Segmenting Retinal Vessels

**DOI:** 10.1155/2022/3585506

**Published:** 2022-08-28

**Authors:** Chen Yue, Mingquan Ye, Peipei Wang, Daobin Huang, Xiaojie Lu

**Affiliations:** ^1^School of Medical Information, Wannan Medical College, Wuhu 241002, China; ^2^Research Center of Health Big Data Mining and Applications, Wannan Medical College, Wuhu 241002, China

## Abstract

This study develops an accurate method based on the generative adversarial network (GAN) that targets the issue of the current discontinuity of micro vessel segmentation in the retinal segmentation images. The processing of images has become increasingly efficient since the advent of deep learning method. We have proposed an improved GAN combined with SE-ResNet and dilated inception block for the segmenting retinal vessels (SAD-GAN). The GAN model has been improved with respect to the following points. (1) In the generator, the original convolution block is replaced with SE-ResNet module. Furthermore, SE-Net can extract the global channel information, while concomitantly strengthening and weakening the key features and invalid features, respectively. The residual structure can alleviate the issue of gradient disappearance. (2) The inception block and dilated convolution are introduced into the discriminator, which enhance the transmission of features and expand the acceptance domain for improved extraction of the deep network features. (3) We have included the attention mechanism in the discriminator for combining the local features with the corresponding global dependencies, and for highlighting the interdependent channel mapping. SAD-GAN performs satisfactorily on public retina datasets. On DRIVE dataset, ROC_AUC and PR_AUC reach 0.9813 and 0.8928, respectively. On CHASE_DB1 dataset, ROC_AUC and PR_AUC reach 0.9839 and 0.9002, respectively. Experimental results demonstrate that the generative adversarial model, combined with deep convolutional neural network, enhances the segmentation accuracy of the retinal vessels far above that of certain state-of-the-art methods.

## 1. Introduction

**Figure 1:**
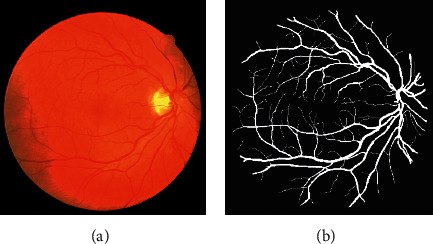
The retinal blood vessel images. (a) Fundus image. (b) Ground truth.

We can infer the vascular tissue condition of the whole body by examining the retinal blood vessels of the fundus. Since there are several small capillary structures in the fundus, as shown in Figure 1, the manual segmentation of the retinal blood vessel images is complicated and cumbersome. Recently, computer vision technology has been extensively employed in image classification, target tracking [1, 2], semantic segmentation, and other fields. Therefore, scholars have proposed certain efficient automatic segmentation algorithms for the retinal blood vessels, which can assist doctors to complete the segmentation and diagnosis with the aid of computers. Traditional image segmentation methods are based on edge detection [3], threshold [4], and region [5] segmentation methods. However, since medical images tend to have complex textures and blurred boundaries, the abovementioned methods sometimes do not work well.

Recently, the application of the convolutional neural networks in various image scenes has brought breakthroughs in several fields of image processing, such as image classification [6] and semantic segmentation [7]. The capability of the automatic feature extraction of the deep learning algorithms effectively overcomes the drawbacks of the traditional medical image segmentation algorithms that exceedingly rely on the prior cognition of the medical experts. Furthermore, the deep learning algorithms are highly portable and can be quickly expanded to different task scenarios with the help of transfer learning.

The GAN model is one of the most promising methods for unsupervised and semi-supervised learning [8–10]. However, the semi-supervised methods mandate a large number of unlabeled data sets for the experiments. According to the previous studies, the experiments are complex, hence supervised learning is still considered in this paper. Luc et al. [11] have used adversarial training for semantic segmentation and proved the accuracy of the method on publicly available data sets. Xue et al. [12] have proposed the first adversarial network SegAN for medical image segmentation. SegAN has taken a fully convolutional neural network as a segment and introduced a multi-scale L1 loss function to evaluate the segmentation results, thus obtaining a higher segmentation accuracy. Isola et al. [13] have proposed a general framework of “image-to-image conversion,” which solves the problems of conversion and loss function with respect to different types of images, in the traditional methods. The generator of DR-GAN, proposed by Zhou et al. [14], takes the retinal structure, pathology, and adaptive grading vectors as the conditions, and introduces a multi-scale space and channel attention mechanism to extract effective feature information. The RV-GAN proposed by Kamran et al. [15] has used multiple generators and multi-scale discriminators and introduced a new loss function that can improve the segmentation accuracy of the retinal thick and thin blood vessels. The GAN proposed by Tavakkoli et al. [16] can generate the fluorescein angiography (FA) images from the fundus photos with a high segmentation accuracy, with a quality that exceeds that of the images from the most advanced algorithms.

**Figure 2:**
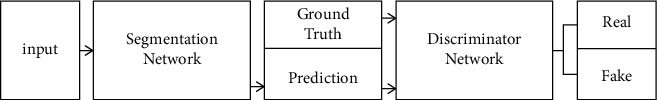
Overall architecture of SAD-GAN.

The model proposed in this paper, termed SAD-GAN, SAD-GAN is composed of a generator and a discriminator, as shown in Figure 2. The fundus images are input to the generator. Following the generation of the segmentation maps, they are input into the discriminator along with the label images, and the discriminator distinguishes the generated segmentation images from the labeled ones. The discriminator shares the parameters with the generator, and both play a game against each other. The segmentation effect of the generator is improved through continuously alternating iterative training.

Although most of the studies demonstrate that the GAN performs well in image segmentation, its segmentation accuracy needs improvement, since the adversarial learning of the discriminator in the GAN affects the segmentation accuracy of the final generator. Therefore, it is essential to design a discriminator that can accurately distinguish the prediction graph from the ground truth. Furthermore, the design of the loss function in the adversarial training also affects the segmentation result. Therefore, we optimize the generator and discriminator by using the convolutional neural network and add the adversarial loss function to the cross-entropy loss function to improve the segmentation result, by controlling the weight.

## 2. Related Works

Since Ronneberger et al. [17] have proposed U-Net, U-Net has been extensively used in image segmentation. Owing to its unique encoding and decoding structure, the performance of the image segmentation has greatly improved, hence it is extensively applied in computer vision research. However, as the network level deepens, the problem of the gradient disappearance easily occurs, and the emergence of residual learning and dense connections can alleviate this problem. For example, Ding et al. [18] have introduced the residual learning, feature fusion, and balance modules in the proposed MRU-Net, and achieved high accuracy on the retinal data set. Alom et al. [19] have proposed a cyclic residual model based on U-Net that can extract the effective segmentation features and segmentation performance of the network, and it achieved good segmentation results. Ibtehaz and Rahman [20] have proposed MultiResUNet, which employed the residual structure in the encoding stage, decoding stage, and skip connection part, and henceforth, the performance relatively improved for the multiple datasets. The MPS-Net proposed by Lin et al. [21] adopts one high-resolution and two low-resolution routes. The proposed multi-path scale module enhances the representation ability of the model, and the significantly improved multiple indicators confirm that the method has good application prospects. The DCU-Net proposed by Yang et al. [22] is a segmentation model that connects the two U-Nets. DCU-Net is primarily composed of the residual deformable convolution modules to enhance the feature extraction ability of the model. To improve the information communication between the two U-shaped networks, a residual attention module has been designed to connect the two U-shaped networks, which achieves a high segmentation accuracy on the retina datasets. Zhai et al. [23] have adopted the inception module in the encoder to obtain the multi-scale features and adopted the pyramid pooling module in the decoder to fuse the multi-scale information. Experiments show that this method improves the segmentation effect.

Once the model has excessive parameters, it will cause an information overload. However, the attention mechanism can focus on the key information and obtain the target area, besides suppressing or filtering out the irrelevant information interference and improving the efficiency of the computing processing. The SE-Net proposed by Hu et al. [24] has focused on the channel of the feature map and then multiplied it with the original feature map. The convolutional block attention module (CBAM) proposed by Woo et al. [25] is a simple and effective feedforward convolutional neural network attention module. CBAM applies attention to both the channel and spatial dimensions. CBAM has the same advantages as a SE module. They can be applied to most models, thus making the model more capable of extracting the feature information without complicating the model. The attention mechanism is employed multiple times in this article. Recently, most of the relevant works have demonstrated that the attention mechanism can enhance feature expression. According to several studies, we can see the significance of the attention mechanism and its wide range of applications. For example, Li et al. [26] have proposed a module that combines a triple attention mechanism, channel, space, and feature internal attention mechanism to improve the correlation between feature maps of various dimensions. Wang et al. [27] have proposed a residual attention network combined with a feedforward network system. The different perception functions of attention modules have been altered through the depth of stacking, and they have achieved a good target recognition on three different datasets. On the basis of FCN, Fu et al. [28] have adopted a dual attention network, channel attention, and spatial attention to obtaining the characteristics of the related dimensions. Furthermore, it has achieved a high segmentation performance on three different scene datasets. Wang et al. [29] have proposed an effective channel attention module, and through the local cross-channel interaction method, the dimensionality reduction was avoided and the parameters were reduced, besides effectively reducing the complexity of the model. Huang et al. [30] have proposed a cross-network CCNet. The proposed cyclic cross-attention module has collected the pixel information on the cross paths and captured the image dependencies. The proposed method has reduced the GPU memory usage and demonstrated a high computational efficiency. The DAR-Net proposed by Li and Wang [31] has used a dense residual module, and it added the channel and spatial attention to the module to select the feature information in the feature map that performed well on the multiple data sets. The DCACNet proposed by Lu et al. [32] has also applied the dual attention mechanism to the medical image segmentation, thus establishing the pixel associations and adding feature representations.

Since Radford et al. [33] have proposed the deep convolutional generative adversarial networks, which can combine the deep convolutional neural network with the generative adversarial network, this method is more rapid and stable in the experiment with an excellent effect. Although the generative adversarial networks are extensively used in the field of image processing, they are rarely seen in retinal image processing [34]. Therefore, this work focuses on the combination of the generative adversarial network and the deep convolutional neural network.

The main contributions from our work can be listed as follows: (1) We have proposed a module that combines ResNet and SE-Net in SAD-GAN. The module is employed as the convolutional layer of the generator. Residual connections can alleviate the gradient disappearance and improve computational efficiency. The attention mechanism can extract the global effective information, and suppress the interference of redundant information. (2) We have added a module in SAD-GAN that combines the dilated convolution and inception in the discriminator. For extracting the effective features, we have used the dilated convolution to obtain the multi-scale features through different dilation rates. Furthermore, we have obtained a larger receptive field and context information by capturing the global information. We employ the Inception-ResNet structure proposed by Szegedy et al. [35] to solve the problems of excessive calculation parameters and disappearance of gradients, which can reduce the redundant calculations through the inception structure and alleviate the phenomenon of the gradient disappearance by adding the residual connections. Furthermore, the structure improves the utilization of the computing resources. We have included the attention mechanism at the end of the discriminator to obtain spatial attention and channel attention to improve the classification accuracy of the discriminator.

## 3. Methods

### 3.1. Generator

**Figure 3:**
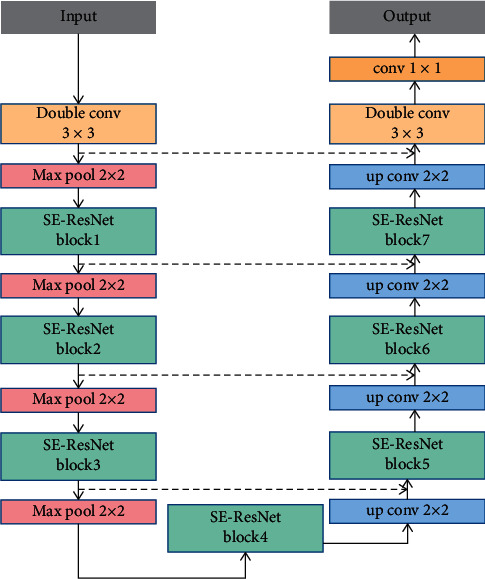
The structure of the generator.

The generator model used in this work follows the traditional U-shaped structure, as shown in Figure 3. The U-shaped network has been proposed by Ronneberger et al. whose core idea is the encoding and decoding structures. The function of the encoding structure is to obtain the feature information of the image and expand the acceptance domain through the pooling operation. The decoding structure locates and classifies the pixels, which allows the network to propagate the contextual information to higher resolution levels, and the skip connection combines the encoding layer features with the decoding layer features to reduce the loss of information and achieve an effective image segmentation.

To improve the segmentation accuracy, we have employed an improved convolution layer, which combines the residual structure and SE-Net, termed SE-ResNet block. The generator model employed in this work consists of two traditional convolution layers, seven SE-ResNet blocks, four max-pooling layers, four up sampling layers, and a conv 1 × 1 layer. Subsequent to 1 × 1 convolution, the pixels are divided into two categories and the segmented image is output.

In the original U-Net model, the traditional convolution layer is composed of two Conv-BN-ReLU. Frequent convolution and pooling operations result in losing a lot of feature information. Residual structure can retain the feature mapping in the deep network, thus alleviating the risk of the gradient message and easing the optimization of the model. Batch normalization (BN) method normalizes the activation values of the hidden layer neurons to prevent the gradient disappearance and improve the model expression ability. ReLU can reduce the model complexity and speed up the convergence.

**Figure 4:**
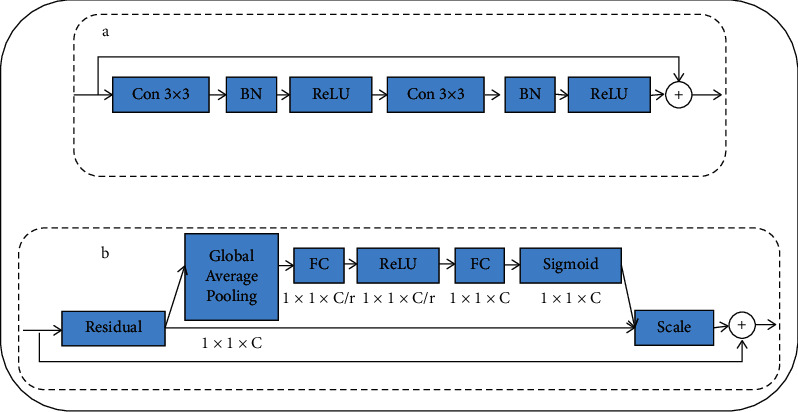
SE-ResNet module combines residual block and SE-Net. (a) Residual block. (b) SE-Net module.

SE-ResNet module combines the residual block and SE-Net. SE-Net module is an attention mechanism that extracts channel information, emphasizes global effective information features, and inhibits the irrelevant information features, which can alleviate the risk of deep network overfitting. In Figure 4, SE-Net consists of five continuous functions, viz., global average pooling, fully connected (FC) layer, ReLU, fully connected (FC) layer, and sigmoid function. Furthermore, *C* is the channel feature number, *r* is the reduction ratio that is set to 2. The global average pooling can extract the global feature information, after a fully connected layer. The channel feature number is compressed into *C*/*r*, through ReLU, to improve the nonlinear. Following another fully connected layer, the number of the channel features is restored to *C*, and the sigmoid function can obtain the weight of each channel. Finally, the features of each channel are multiplied by the corresponding weights as the output. SE-Net can be used along with any architecture.

### 3.2. Discriminator

**Figure 5:**
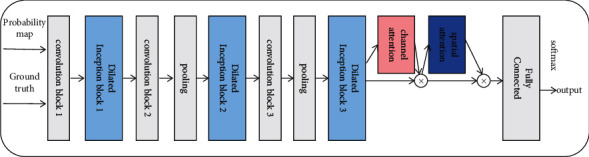
The structure of the discriminator.

**Figure 6:**
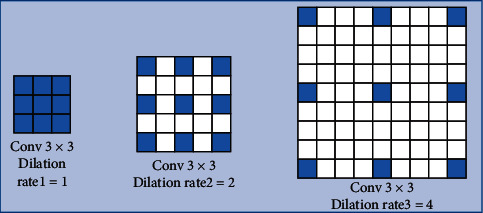
The dilated convolution with the different dilation rates.

The structure of the discriminator is displayed in Figure 5. The structure of the first convolution block is Conv 3 × 3-BN-ReLU, and the output of the first convolution block is used as the input of the inception block. We have used the Inception-ResNet proposed by Szegedy et al. [35]. Inception module has for long been extensively used. It has the advantages of extracting the multi-scale features, separating spatial features, and selecting the channel information. In this work, the combination of the inception module and dilated convolution can reduce the number of parameters and expand the acceptance field. In Figure 6, the values of rates 1, 2, and 3, are 1, 2, and 4, respectively. Thereafter, it passes through the down sampling layer composed of the convolution and pooling layers. The second and third convolution blocks are Conv 1 × 1-BN-ReLU, which reduce the dimension of the channel number, number of parameters, and the risk of network overfitting while improving the nonlinear characteristics and accelerating the training process. The pooling layer is composed of the average pooling 2 × 2. The reason for the adoption of the average pooling instead of max pooling is that in the image classification, the average pooling can retain the high-level semantic information in the deep network and reduce information loss. Furthermore, the size of feature maps can be changed through convolution and pooling operations. This discriminator introduces the channel and spatial attentions to combine the local and global information, thus reducing the redundancy of information in the physical signs and extracting more effective information. Through a series of operations, the sample features can be extracted. Finally, it distinguishes the ground truth and the generated retinal blood vessel segmentation images through the fully connected and SoftMax.

#### 3.2.1. Dilated Inception Block

**Figure 7:**
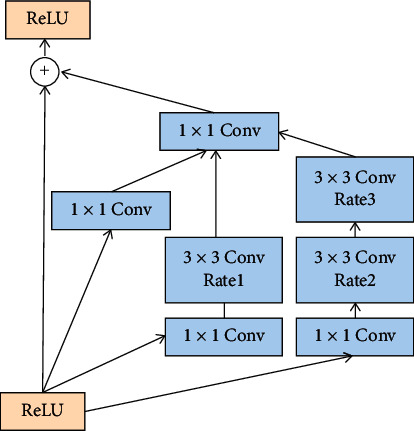
The dilated inception block.

In a convolutional neural network, different convolution kernels represent different receptive fields, and the convolution kernel is larger for a larger feature scale. However, excessively large convolution kernels often expand the number of parameters, thus resulting in heavy computing tasks. Therefore, in the previous studies, the method of replacing the large convolution kernels with dilated convolution has appeared. The dilated convolution can obtain the acceptance domain of different scales, besides saving a lot of computation [36]. In Figure 6, we have used the dilated convolution with the dilation rates of 1, 2, and 4, thus the acceptance field can be expanded to obtain features of different scales. The inception has been proposed earlier and is widely used in various computer vision studies. Its variant plays an important role in the deep network [37]. Therefore, according to Figure 7, we combine inception and dilated convolution to extract the multi-scale features with fewer parameters to improve the overall performance of the network.

#### 3.2.2. Attention Mechanism

**Figure 8:**
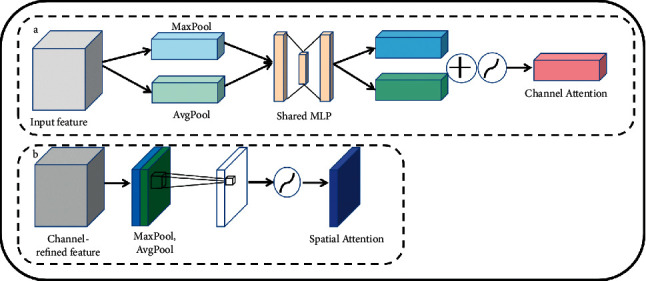
The attention mechanism. (a) Channel attention module. (b) Spatial attention module.

According to Figure 8(a), the channel attention focuses on the channel dimension. Each channel of the feature map has been considered as a feature detected by a feature detector, and each channel feature is obtained by a different convolution kernel. For a feature map (*C* × *H* × *W*), we have calculated the importance of each channel, which is the weight (*C* × 1 × 1). Furthermore, we multiply the weight with the feature map to obtain a weighted feature map, which gives the channel attention. The weight is calculated by MaxPool and AvgPool. To obtain the weight of the channel dimension, each channel (1 × *H* × *W*) needs to be compressed into a number (1 × 1 × 1). The two methods are given in the following descriptions. We have calculated the average value (AvgPool) and the maximum value of each channel (MaxPool). Furthermore, we pass them through a common multilayer perceptron MLP. The structure of MLP is an input or the input neuron *C*, and the hidden layer neuron *C*/*r*, besides the output neuron *C*. Thereafter, we add these two outputs and normalize them with the sigmoid function to get the final weight. Then we multiply this weight with the original feature map to get the weighted feature map in the channel dimension.

Spatial attention focuses on “where,” which is an important issue in each of the channels. Its purpose is to perform AvgPool and MaxPool operations on different channel values on the same plane space point and pass a convolutional layer and a sigmoid function to obtain the weight. Furthermore, we multiply this weight with each channel in the spatial dimension, and finally, get the weighted feature map in the spatial dimension. The spatial attention module is shown in Figure 8(b). It can simulate the rich global feature context information so that the same feature in different locations can be enhanced. Furthermore, the semantic segmentation ability can be improved. In the last part, we have used the sequential model, and let the feature map pass the channel attention first, and then pass the spatial attention.

### 3.3. Loss Function

Formula (1) is the loss function of the discriminator, which is the minimum spatial cross-entropy loss function for two classes.

(1) $$\begin{array}{c} L_{D}=-\sum_{h,w}\left(\left(1-q\right)\cdot \;\log\left(1-D{\left(S\left(x\right)\right)}^{\left(h,w\right)}\right)\right)+\left(q\cdot \;\log\left(D{\left(y\right)}^{\left(h,w\right)}\right)\right). \end{array}$$

When *q* is zero, the sample comes from the generator, and when *q* is 1, it comes from the ground truth. Here, *x* represents the input image, *y* represents the ground truth, and *y*(*h*, *w*) represents the pixel value of *y* at the position (*h*, *w*). Formula (2), given below, is the loss function of the generator, which is composed of the cross entropy and adversarial losses.

(2) $$\begin{array}{c} L_{\text{seg}}=L_{\text{ce}}+\lambda_{\text{adv}}L_{\text{adv}}, \end{array}$$

*λ*_adv_ is the weight to minimize *L*_seg_. The cross-entropy loss is shown in equation (3).

(3) $$\begin{array}{c} L_{\text{ce}}=-\sum_{h,w}\sum_{c\in C}y^{\left(h,w,c\right)}\log\left(S{\left(x\right)}^{\left(h,w,c\right)}\right), \end{array}$$

where *x* is the input image, *y* is the ground truth, *S*(*x*) is the prediction result, and *C* refers to the number of classes. Formula (4) is the adversarial loss. To train the generator and trick the discriminator, we have added adversarial loss to maximize the probability of the predicted results being generated from the ground truth distribution.

(4) $$\begin{array}{c} L_{\text{adv}}=-\sum_{h,w}\log\left(D\left(S^{\left(h,w\right)}\left(x\right)\right)\right). \end{array}$$

## 4. Results and Discussion

To demonstrate the performance of the SAD-GAN model, we have tested them on two retinal image datasets, viz., DRIVE [38] and CHASE_DB1 [39]. We have employed Adam optimizer for both the networks and we trained the generator with a weight decay = 10^−4^, learning rate = 2.5 × 10^−4^, and learning rate of discriminator = 10^−4^. Furthermore, we have set *λ*_adv_ to 0.1, batch_size = 1, and the number of iterations of the two data sets to 20000. To complete the implementation, we have used the PyTorch and TensorFlow frameworks on a single GPU machine with 16G RAM and NVIDIA GEFORCE GTX-1650 SUPER.

### 4.1. Database and Preprocessing Methods

Database: The DRIVE dataset includes 40 retinal images. The size of each image is 584 × 565. The CHASE_DB1 dataset includes 28 retinal images. The size of each image is 999 × 960. Both the datasets have the mask and ground truth manually segmented by experts.

Preprocessing: Random rotation and mirror flip have been employed to increase the number of datasets. Since the contrast between the G channel and the blood vessel is the highest among the three RGB channels, we have extracted the G channel to obtain the grayscale image, and then normalized it. Furthermore, we have used the contrast-limited adaptive histogram equalization (CLAHE), to improve the contrast between the blood vessel and the background. Finally, the gamma transform algorithm has been employed to increase the brightness of the darker part without affecting the brighter part. The final data set is partitioned as 10% for validation and 90% for training.

### 4.2. Evaluation Indexes

Retinal blood vessel segmentation is intended to divide the pixels in the color retinal image into the blood vessels and background. This work has employed accuracy (ACC), sensitivity (SE), specificity (SP), ROC_AUC, and PR_AUC to judge the effectiveness of the retinal blood vessel segmentation method in this work. These indices are the five commonly used ones to measure the performance of the algorithm. Their calculation methods are listed in Table 1. ROC_AUC represents the relationship between sensitivity and specificity, whereas ROC_AUC and PR_AUC represent the prediction accuracy of the image segmentation result. The segmentation effect of the algorithm is better for the value of AUC proximate to one.

### 4.3. Ablation Study

**Figure 9:**
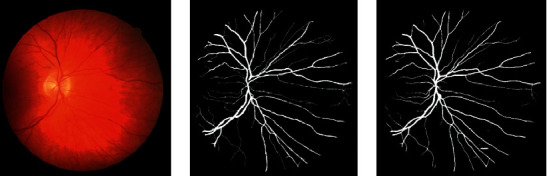
From left to right are the fundus image, ground truth, and the segmentation result of SAD-GAN on DRIVE, respectively.

**Figure 10:**
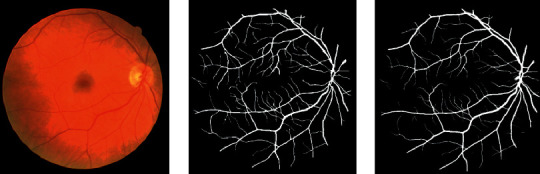
From left to right are the fundus image, ground truth, and the segmentation result of SAD-GAN on DRIVE, respectively.

**Figure 11:**
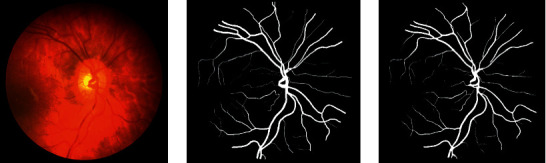
From left to right are the fundus image, ground truth, and the segmentation result of SAD-GAN on CHASE_DB1, respectively.

**Figure 12:**
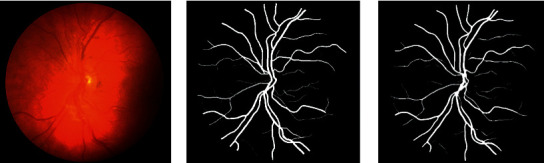
From left to right are the fundus image, ground truth, and the segmentation result of SAD-GAN on CHASE_DB1, respectively.

Figures 9–12 are the segmentation results of SAD-GAN on DRIVE and CHASE_DB1. We have conducted comparative experiments to ensure that the generator and discriminator, which we have proposed, to improve the segmentation accuracy. SD-GAN: the generator is identical to SAD-GAN, and we replace the inception module in the discriminator with a standard convolution layer consisting of two Conv 3 × 3-BN-ReLU. Furthermore, we remove the attention module, and this model is called SD-GAN. RA-GAN: the generator is identical to that of SAD-GAN, and the discriminator removes the attention module from the discriminator of SAD-GAN, and this model is named as RA-GAN. SG-GAN: we replace the SE-ResNet block in the generator with two Conv 3 × 3-BN-ReLU. Furthermore, the discriminator is identical to SAD-GAN, and this model is termed SG-GAN. We have used SAD-GAN, SD-GAN, RA-GAN, and SG-GAN models to conduct the experiments.

**Figure 13:**
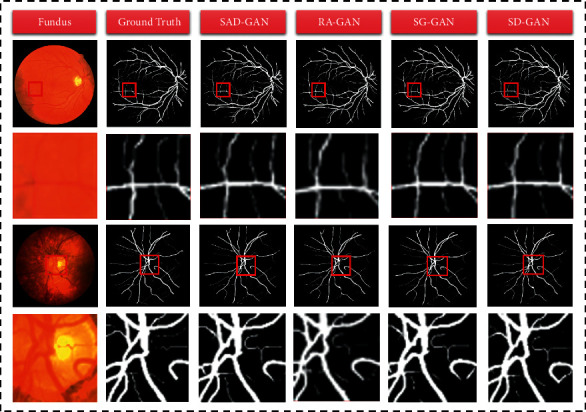
The first two lines and the last two lines are detailed segmentation diagrams of different GAN models on DRIVE and CHASE_DB1, respectively.

To further demonstrate the effectiveness of the improvement measures, this work has employed four different GAN models to conduct experiments on the DRIVE and CHASE_DB1 datasets. The experimental results are displayed in Figure 13. The first to sixth columns represent the original image, ground truth, segmentation result of SAD-GAN, segmentation result of the RA-GAN model, segmentation result of the SG-GAN model, and segmentation result of the SD-GAN model, respectively. We have found through comparison that the SAD-GAN network structure excels other network models in the overall performance of both datasets. The segmentation results of the algorithm in this paper are consistent with the expert manual segmentation results. Compared with other algorithms, the SAD-GAN model employed in this paper can better overcome the difficulties in retinal segmentation and achieve better segmentation results for small blood vessels.

#### 4.3.1. Comparison of the Segmentation Indicators of Different GAN Algorithms in this Paper

**Figure 14:**
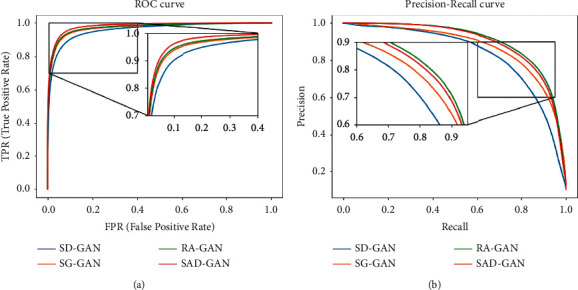
The curves of four GAN models. (a) ROC curves for DRIVE dataset. (b) PR curves for DRIVE dataset.

**Figure 15:**
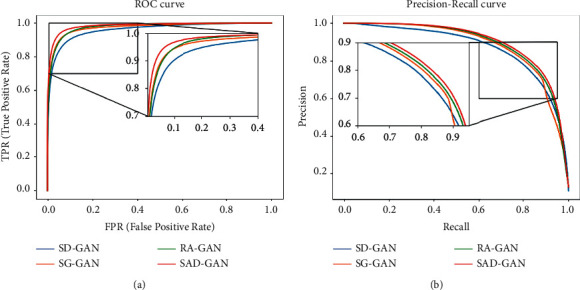
The curves of four GAN models. (a) ROC curves for CHASE_DB1 dataset. (b) PR curves for CHASE_DB1 dataset.

Figures 14 and 15 show the ROC and PR curves, respectively, of several types of models under the DRIVE and CHASE_DB1 datasets. When compared with RA-GAN, SG-GAN, and SD-GAN, the PR_AUC value of RA-GAN is 0.9003 on DRIVE, which exceeds that of SAD-GAN. However, SAD-GAN has the largest area under ROC on both datasets and has the largest area under PR on the CHASE_DB1 dataset. Hence, its segmentation performance is relatively better. The segmentation results of SG-GAN, SD-GAN, RA-GAN, and SAD-GAN have been compared. The results of the experiments are listed in Tables 2 and 3. On the DRIVE dataset, the ACC value of the algorithm in this work is 0.9567, the SE value is 0.8422, the SP value is 0.9825, the ROC_AUC value is 0.9813, and the PR_AUC value is 0.8928. On the CHASE_DB1 dataset, the ACC value of the algorithm given in this work is 0.9671, the SE value is 0.8503, the SP value is 0.9850, the ROC_AUC value is 0.9839, and the PR_AUC value is 0.9002. Compared with the first three models, the recognition accuracy of SAD-GAN is higher, which demonstrates that SAD-GAN can segment the retinal vascular structure accurately. According to the tables, the indicators of SAD-GAN exceed those of the other three models, which establishes that the improved generator and discriminator proposed in this paper have marked effects, thus tremendously improving the overall performance and the network prediction ability.

#### 4.3.2. Comparison of the Segmentation Indicators between the Algorithm in this Article and Other Algorithms

Tables 4 and 5 display the index results of SAD-GAN and recent retinal segmentation methods on DRIVE and CHASE_DB1 datasets. SUD-GAN proposed by Yang et al. [46] is also a generative adversarial network for retinal vascular segmentation. On the DRIVE dataset, the algorithm proposed in this work outperforms SUD-GAN in all indicators. Furthermore, PR_AUC has been improved by nearly 10%, whereas PR_AUC is 0.8928 and sensitivity is 0.8422. ROC_AUC is 0.9813, which is the highest value in Table 4. On the CHASE_DB1 dataset, the accuracy, specificity, and PR_AUC have been significantly improved, which are 0.9671, 0.9850, and 0.9002, respectively. According to Table 5, the index results of the algorithm proposed in this paper are not inferior to other algorithms. Particularly, the AUC indices exceed that of most of the algorithms listed in the tables. Therefore, the method proposed in this paper is accurate and robust for the segmentation of tiny retinal vessels.

## 5. Conclusions

Regular retinal blood vessel detection can timely detect vascular abnormalities, help patients to advance treatment, and prevent the further development and deterioration of the disease. Employing artificial intelligence to assist doctors to segment the retinal blood vessels has practical significance for rapid diagnosis. We propose an improved generative adversarial network for retinal image segmentation, which is intended to solve the problems of inaccurate and discontinuous retinal blood vessel segmentation. In the generator, we have introduced the residual structure and SE-Net to extract the global channel feature information without significantly increasing the number of parameters, which avoids overfitting and alleviation of the phenomenon of gradient disappearance, thus improving the segmentation accuracy. In the discriminator, we have replaced the standard convolution layer with the inception and dilated convolution layer, which can extract the high-level semantic information of different scale features of the deep network. This is important for accurately distinguishing the prediction maps from the ground truth. The channel attention and spatial attention that we have introduced also play an important role in the feature extraction, thus reducing the influence of the redundant information in the image classification by focusing on the useful information. By testing on the public datasets DRIVE and CHASE_DB1, our method achieves sensitivity = 0.8422, specificity = 0.9825, accuracy = 0.9567, ROC_AUC = 0.9813, PR_AUC = 0.8928 on the DRIVE dataset, whereas the sensitivity = 0.8503, specificity = 0.9850, accuracy = 0.9671, ROC_AUC = 0.9839, and PR_AUC = 0.9002 on the CHASE_DB1 dataset. The algorithm in this work has high accuracy and excels in some of the current state-of-the-art methods. It can segment the small retinal blood vessels better. The adversarial loss function that we have included plays a certain role in improving the segmentation accuracy in the overall training. We will further discuss the appropriate loss functions in future work on the experimental designs.

## Figures and Tables

**Table 1:** The evaluation indexes.

Evaluation metric	Description
Accuracy (ACC)	(*P*_TP_+*P*_TN_)/(*P*_TP_+*P*_FP_+*P*_FN_+*P*_TN_)
Sensitivity (SE)	*P*_TP_/(*P*_TP_+*P*_FN_)
Specificity (SP)	*P*_TN_/(*P*_TN_+*P*_FP_)

**Table 2:** The following table shows the segmentation results of different GAN models on DRIVE dataset in this paper.

Methods	SE	SP	ACC	ROC_AUC	PR_AUC
SD-GAN	0.7782	0.9811	0.9542	0.9585	0.8322
SG-GAN	0.7812	0.9779	0.9553	0.9711	0.8694
RA-GAN	0.8276	0.9802	0.9548	0.9737	0.9003
SAD-GAN	0.8422	0.9825	0.9567	0.9813	0.8928

**Table 3:** The following table shows the segmentation results of different GAN models on CHASE_DB1 dataset in this paper.

Methods	SE	SP	ACC	ROC_AUC	PR_AUC
SD-GAN	0.7893	0.9799	0.9646	0.9594	0.8698
SG-GAN	0.7939	0.9825	0.9651	0.9736	0.8829
RA-GAN	0.8126	0.9834	0.9648	0.9767	0.8927
SAD-GAN	0.8503	0.9850	0.9671	0.9839	0.9002

**Table 4:** The results of SAD-GAN and other algorithms on DRIVE dataset.

Methods	Year	SE	SP	ACC	ROC_AUC	PR_AUC
Wu et al. [40]	2018	0.7844	0.9819	0.9567	0.9807	—
Wang et al. [41]	2019	0.7940	0.9816	0.9567	0.9772	—
Cheng et al. [42]	2019	0.7672	0.9834	0.9559	0.9793	—
Yue et al. [43]	2019	0.8199	0.9762	0.9561	0.9796	—
Li et al. [44]	2020	0.7890	0.9799	0.9557	0.9774	0.8519
Ma et al. [45]	2020	0.7875	0.9813	0.9566	0.9794	—
Yang et al. [46]	2020	0.8340	0.9820	0.9560	0.9786	0.8821
Samuel and Veeramalai [47]	2020	0.7827	0.9821	0.9627	0.9789	
Li et al. [48]	2021	0.7931	**0.9896**	**0.9698**	0.9738	—
Xu et al. [49]	2021	0.8320	0.9885	0.9590	0.9713	
Lin et al. [21]	2021	0.8361	0.9740	0.9563	0.9805	
Yang et al. [22]	2022	0.8115	0.9780	0.9568	0.9810	—
Zhai et al. [23]	2022	0.7982	0.9818	0.9571	0.9811	—
Our proposed		**0.8422**	0.9825	0.9567	**0.9813**	**0.8928**

**Table 5:** The results of SAD-GAN and other algorithms on CHASE_DB1 dataset.

Methods	Year	SE	SP	ACC	ROC_AUC	PR_AUC
Wu et al. [40]	2018	0.7538	0.9847	0.9637	0.9825	—
Wang et al. [41]	2019	0.8074	0.9821	0.9661	0.9812	—
Cheng et al. [42]	2019	**0.8967**	0.9540	0.9488	0.9785	—
Li et al. [44]	2020	0.7798	0.9822	0.9620	0.9791	0.8291
Lin et al. [21]	2021	0.8488	0.9795	0.9668	0.9869	
Yang et al. [22]	2022	0.8075	0.9841	0.9664	**0.9872**	—
Our proposed		0.8503	**0.9850**	**0.9671**	0.9839	**0.9002**

## Data Availability

The data used to support the findings of this study are available from the corresponding author upon request.
